# Oral Health-Related Quality of Life and Mental Health Impairment in Patients Affected by Medication-Related Osteonecrosis of the Jaws: A Case–Control Pilot Study

**DOI:** 10.3390/dj11060147

**Published:** 2023-06-07

**Authors:** Elena Calabria, Alessandro Antonelli, Selene Barone, Daniela Adamo, Marianna Salviati, Maria Giulia Cerra, Francesco Bennardo, Amerigo Giudice

**Affiliations:** 1 Department of Health Sciences, School of Dentistry, Magna Graecia University of Catanzaro, 88100 Catanzaro, Italy; calabriaelena92@gmail.com (E.C.); selene.barone@unicz.it (S.B.); mariannasalviati97@gmail.com (M.S.); m.giulia223@gmail.com (M.G.C.); francesco.bennardo@unicz.it (F.B.); a.giudice@unicz.it (A.G.); 2 Department of Neuroscience, Reproductive Science and Dentistry, University of Naples “Federico II”, 80131 Naples, Italy; danielaadamo.it@gmail.com

**Keywords:** medication-related osteonecrosis of the jaws, anxiety, depression, quality of life, oral health-related quality of life

## Abstract

In the present case–control study, the impact of medication-related osteonecrosis of the jaws (MRONJ) on patients’ oral health-related quality of life (OHRQoL), overall quality of life (QoL), and psychological status was evaluated using a set of questionnaires. These questionnaires included the Oral Health Impact Profile-14 (OHIP-14), the Short Form 36 Health Survey Questionnaire (SF-36), and the hospital anxiety and depression scale (HADS). A total of 25 MRONJ patients and 25 controls were included in the study. The results showed that MRONJ patients had a significantly poorer OHRQoL (OHIP-14 score *p*-value: 0.003) and lower general QoL, particularly in the domains of “physical functioning”, “physical role”, “body pain”, “general health”, and “vitality” in the SF-36 questionnaire (*p*-values: 0.001, 0.001, 0.013, 0.001, and 0.020). Although there were no significant differences between the groups in the SF-36 domains of “social functioning”, “emotional role”, and “mental health”, the mean sub-scores of the HADS, specifically the depression and anxiety scores (HADS-D and HADS-A), were significantly higher in MRONJ patients (*p*-values: 0.002 and 0.009). However, the “mental health” domain of the SF-36 questionnaire showed a correlation with both HADS-A and HADS-D scores (*p*-values: 0.003 and 0.031). Therefore, a comprehensive clinical examination of MRONJ patients should include the assessment of OHRQoL, overall QoL, and psychological profile using different questionnaires. This approach aims to gather detailed information about patients’ physical and psychological well-being, enabling the development of tailored treatments.

## 1. Introduction

Medication-related osteonecrosis of the jaw (MRONJ) is an adverse event that may develop in the oral cavity of patients currently or previously treated with anti-resorptive therapy alone or combined with immunomodulators or anti-angiogenic drugs, in the absence of prior radiotherapy of the head–neck area [1]. MRONJ usually involves oncological patients with bone metastases or who are affected by severe osteoporosis and, therefore, at a higher risk of developing skeletal-related events (SRE) [2]. For this reason, while the use of anti-resorptive drugs such as Denosumab or bisphosphonates can improve the quality of life (QoL) of these patients, they also expose them to the risk of developing MRONJ [3]. Indeed, although the use of these drugs may cause rare but serious toxicities such as MRONJ, there remains a strong rationale for the clinical utility of anti-resorptive therapy for the prevention of SRE by improving patients’ QoL [4]. However, the general medical conditions of these patients are very poor, and the onset of MRONJ could further negatively influence the health condition and worsen the patients’ QoL [5,6].

MRONJ may have a difficult course resulting in tooth loss and altered function, further negatively affecting patients’ eating, physical appearance, speech, breathing, and swallowing [7,8], therefore severely impacting their oral health-related quality of life (OHQoL) [9,10]. Additionally, painful symptoms and recurrent jawbone infections can severely impair the patient’s psychological aspect [10,11]. Moreover, although many international guidelines and clinical recommendations on the management of MRONJ are available, the most appropriate treatment is a matter of debate among researchers and clinicians [1,12,13,14]. Indeed, although cases of spontaneous resolution of MRONJ have been reported in the literature, some authors showed that conservative therapy can help clinicians in the management of the early stages of MRONJ [15,16,17,18]. 

While the American Association of Oral and Maxillofacial Surgeons (AAOMS) Position Paper has confirmed that one of the goals of MRONJ treatment is to preserve the QoL of the patient [1], there are still few studies that have introduced this parameter among the traditional outcomes [19,20,21]. Indeed, the majority of the current studies have used downstaging, resolution of painful symptoms or infection, and mucosal coverage as the outcomes of interest in MRONJ management [22]. However, these outcomes may be insufficient in guiding oral health providers to the best therapy and results, as the general health and psychological status of the patients are not evaluated [23]. Moreover, there is still no consensus on which set of questionnaires may be more appropriate for assessing the QoL of MRONJ patients. Up to now, only a few studies have investigated the OHRQoL and the general QoL of MRONJ patients using different types of questionnaires [9,24]. Of note, only recently a specific questionnaire assessing the QoL in MRONJ patients has been validated, but it is still available exclusively in English [6].

Further, while there is growing attention to the evaluation of the psychological well-being of patients affected by other chronic oral diseases [25,26,27], to date, the psychological profile of MRONJ patients has not yet been specifically investigated [19,28].

The hypothesis is that MRONJ impacts the OHRQoL of the affected patients, and also influences the general well-being and mental health of the patients. The research question on which the present study was conducted is as follows:

Can MRONJ affect the OHRQoL, QoL, and mental health of MRONJ patients? To what extent are these parameters altered in MRONJ patients in comparison to a control group without MRONJ?

To answer the research question, the primary aim of this study was to conduct an exploratory analysis of the OHRQoL, QoL, and psychological profile, in particular anxiety and depression, of MRONJ patients in comparison to a group of controls matched by gender and age. Finally, the secondary aim was to analyze the correlation between the OHRQoL and QoL with the psychological profile in MRONJ patients. 

## 2. Materials and Methods

### 2.1. Study Design and Participants

A case–control study was designed and carried out at the Oral Pathology and Surgery Department of the University Magna Graecia of Catanzaro, Italy, between September 2022 and December 2022. All the methods adopted were in accordance with the Strengthening of the Reporting of Observational Studies in Epidemiology (STROBE) guidelines for case–control studies [29]. The study was approved by the local Ethics Committee of the same University. All participants of both genders were consecutively enrolled in the dental clinic of the University Magna Graecia of Catanzaro. First, MRONJ patients were enrolled, and their mean age and percentage of females and males were calculated. Then, participants in the control group were recruited in order to obtain two groups matched for age and sex. All the subjects signed written informed consent. MRONJ patients were included according to the following inclusion and exclusion criteria. Inclusion criteria encompassed the following:

- Patients with a confirmed diagnosis of MRONJ formulated according to the AAOMS Position Paper 2022 [1]. Based on these criteria, the case definition of MRONJ includes all the following elements: (i) current or previous treatment with antiresorptive therapy alone or in combination with immunomodulators or anti-angiogenic medications; (ii) exposed bone or bone that can be probed through an intraoral or extraoral fistula(e) in the maxillofacial region that has persisted for more than 8 weeks, and (iii) no history of radiation therapy to the jaws or metastatic disease to the jaws;
- Patients affected by osteometabolic or oncological diseases;
- Patients affected by any MRONJ stage, from stage 0a to 3, according to the AAOMS 2022 staging system;
- Newly diagnosed patients who had not received any treatment for MRONJ at the time of enrollment.
  For the control group, inclusion criteria encompassed the following:
- Participants referred for routine dental care (for instance, restorative or non-surgical periodontal treatments/follow-up visits) to the dental clinic of the same university during the study period;
- Absence of any oral mucosal disease;
- No current or previous history of MRONJ;
- No current or previous history of malignant disease or osteometabolic diseases.

In both groups, participants were excluded in case of previous history or occurrence of psychiatric illness (as defined by the American Psychiatric Association Diagnostic and Statistical Manual of Mental Disorders V, DSM-V), in case the participants were on therapy with systemic psychotropic drugs, or were unable to understand the questionnaires due to cognitive impairment or mental disability.

### 2.2. Procedure and Data Collection 

At the time of the enrolment, each participant underwent a thorough intra-oral and extra-oral examination performed by two oral medicine specialists (EC and AA) and completed the questionnaires administered. All the relevant data were collected and recorded in a predefined Excel template protected by password. Data collection included demographic characteristics, such as gender, age, body mass index (BMI), systemic comorbidities, smoking, and alcohol consumption; data on the oral health status, specifically the DMFT index and the presence or absence of periodontal disease [30] were also recorded in each participant; data on the disease needing the antiresorptive treatment (AR), as osteometabolic or oncological diseases, the type of malignancy, the presence of bone metastasis, type of AR, and the duration (in months) of the AR treatment; and MRONJ clinical data, as site affected (mandible, maxilla, or both) and stage of the MRONJ lesion at the time of the enrollment adjudicated according to the AAOMS staging system [1]. 

### 2.3. Oral Health-Related Quality of Life, Quality of Life, Pain and Psychological Profile Assessment

Preselected questionnaires were administered as their Italian-validated version and completed by each participant. Each questionnaire was then checked in order to avoid missing data. According to the objectives of the present study, the following questionnaires were used:

- The Oral Health Impact Profile-14 (OHIP-14) was used to evaluate the impact of oral health on an individual’s quality of life. It comprises 14 items exploring seven domains of impact: functional limitation (trouble pronouncing words or worsened taste), physical pain (aching in mouth or discomfort eating food), psychological discomfort (feeling self-conscious or tense), physical disability (interrupted meals or poor diet), psychological disability (difficulty relaxing or embarrassment), social disability (irritability or difficulty in doing usual jobs), and handicap (life less satisfying or inability to function). All items are presented with a five-category rating scale ranging from “never” (0) to “hardly ever” (1), “occasionally” (2), “often” (3), and “very often” (4). The total score of the OHIP-14 is obtained by the sum of all sub-scores and ranges from 0 to 56, with higher scores indicating a poorer oral health-related quality of life (OHRQoL), while the score of each of the seven domains is obtained by the sum of the sub-scores of two consecutive items [31].
- The Short Form 36 Health Survey Questionnaire (SF-36) was used to investigate their QoL. It consists of 36 questions exploring 8 domains: physical functioning (limitations in performing physical activities such as bathing or dressing), physical role (limitations in work and other daily activities as a result of physical health), bodily pain (how severe and limiting is the pain), general health (how general personal health is evaluated by the patient), vitality (feeling tired vs. feeling full of energy), social functioning (interference with normal social activities due to physical or emotional problems), emotional role (limitations in work and other daily activities as a result of emotional problems), and mental health (feeling nervous and depressed vs. feeling peaceful, happy and calm). For each item, the score ranges from 0 to 100, with lower scores indicating a worse QoL [32,33].
- The Numeric Rating Scale (NRS-11) is a well-validated instrument for the evaluation of pain intensity whose scale ranges from 0 to 10 (0 = no oral symptoms and 10 = the worst imaginable discomfort). Respondents are asked to report pain intensity in the last 24 h [34].
- The hospital anxiety and depression scale (HADS) was used to assess the psychological profile of the participants. It consists of 14 items, divided into a 7-item anxiety sub-scale (HADS-A) assessing anxiety symptoms (for instance, feelings of tension, fear, worry, and panic) and a 7-item depression subscale (HADS-D) focusing on depressive symptoms, predominantly anhedonia, which is a core symptom of depression. Each item is scored on a 4-point Likert scale (0–3) with a total score ranging from 0 to 21 for each subscale. Higher scores correspond to a higher level of psychological impairment, with cut-off HADS-A and HADS-D scores of 8 or above, which indicates the presence of anxiety and depression [35,36].

### 2.4. Statistical Analysis 

The R software (v. 4.1 2) (Team Rcore, 2016) was employed for the statistical analyses. Normal distribution assessment was preliminarily performed using the Kolmogorov–Smirnov test for normal distribution. For continuous data, descriptive statistics reported mean and standard deviations (SD). Categorical variables were summarized using absolute and percentage frequencies. Bivariate statistical analysis included the Chi-square test for categorical data and Student’s t-test for continuous variables. Wilcoxon test was used for comparison of continuous asymmetric distributions. Linear regression analysis was performed to evaluate any correlation between OHIP-14 (total score and sub-scores) and the scores of NRS, HADS, and SF-36. Similarly, linear regression analysis was also computed for the SF-36 items’ scores in order to test their relationship with the HAD-A and HAD-D sub-scores. Finally, subgroup analysis was conducted to detect any difference among MRONJ patients according to their primary disease and the stage of the MRONJ lesion. A *p*-value < 0.05 or 0.01 was considered moderately or strongly significant, respectively. The power analysis was performed by considering 50 subjects with the following input parameters: two tails, effect size d = 0.5, and alpha = 0.05. A power of 93% was determined.

## 3. Results

**Figure 1:**
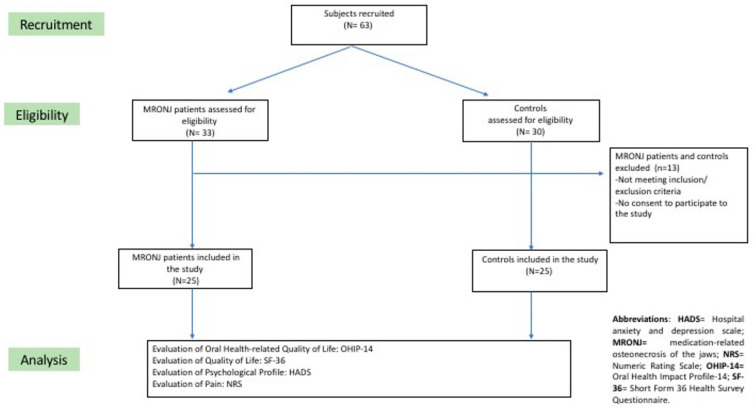
Flow chart of 25 MRONJ patients and 25 controls.

Overall, fifty participants were included: 25 MRONJ patients and 25 controls (Figure 1: flow chart). 

Table 1 shows the socio-demographic profile, BMI, risk factors, and comorbidities of the two samples. In each group, there were 21 females and 4 males, presenting a mean age of 72.3 ± 7.6 years and 71.3 ± 6.9 years in the study group and control group, respectively, with no statistically significant difference (*p*-value: 0.367). The majority of both MRONJ patients (20, 80%) and controls (17, 68.0%) were married (*p*-value: 0.520); however, a statistically higher proportion of MRONJ patients were retired or not employed (21, 88.0%) compared to the controls (12, 48%) (*p*-value: 0.046. In terms of education (expressed in years), BMI, smoking habit, and alcohol consumption, no significant differences were detected between the two groups (*p*-values: 0.071, 0.215, 0.098, and 0.189, respectively). On the contrary, all the MRONJ patients (25, 100%) presented at least one or more systemic comorbidities in addition to the underlying osteometabolic or oncological disease requiring AR treatment, compared to the controls (20, 80.0%) with a moderate statistically significant difference detected (*p*-value: 0.050). However, when comparing the frequency distributions of all the systemic disease categories, no differences were recorded between the two groups.

The clinical characteristics of the MRONJ patients are presented in Table 2. Within this group, 17 (68%) of the patients were affected by an oncological disease requiring the AR treatment: in detail, 12 (48.0%) by breast cancer, 3 (12.0%) by prostate cancer, and 2 (8.0%) by multiple myeloma, whereas 8 (32.0%) patients were affected by primary osteoporosis. With regard to the AR treatment history, at the time of the enrolment, all the patients had already suspended the AR on account of MRONJ suspicion. On average, MRONJ patients had been on AR for a mean of 46.7 ± 44.3 months. The majority of them (14, 56.0%) had received Denosumab, specifically 12 (48%) receiving Xgeva/Denosumab and 2 (8.0%) receiving Prolia/Denosumab. All the others had been on bisphosphonate therapy alone (36%), except for two patients switching to Denosumab therapy after being treated with bisphosphonates (8.0%). In 16 (64.0%) patients, the MRONJ lesion was localized at the mandible, while in 7 (28.0%), it was localized in the maxilla and in 2 (8.0%) cases in both jaws. Sixteen (64.0%) of the MRONJ lesions were adjudicated as stage II at the first consultation, eight (32.0%) as stage I, and only one as stage III, according to the AAOMS staging system. With regard to the oral health status, MRONJ patients presented a statistically significant higher DMFT score compared to the control group (*p*-value: <0.001; mean DMFT score in MRONJ patients = 22.64; and mean DMFT score in control patients = 11.36). In detail, MRONJ presented a higher number of decayed teeth (1.76 vs. 0.8, *p*-value: 0.046), filled teeth (4.76 vs. 1.48, *p*-value: <0.001), and missing teeth (16.0 vs. 9.28, *p*-value: 0.009). In addition, at the time of the enrollment, a statistically significant greater prevalence of periodontal disease was detected in MRONJ patients (23, 92%) in comparison to the control group (13, 52%) (*p*-value: <0.001) (data not displayed in the tables).

The comparison analysis of all the questionnaire scores between the two groups is shown in Table 3. Overall, the mean total score of OHIP-14 was significantly higher in the MRONJ group (21.1 ± 12.7) in comparison to the mean score of the controls (11.0 ± 10.1) (*p*-value: 0.003), meaning that patients affected by MRONJ had a poorer OHRQoL. Specifically, the sub-items of the OHIP-14 whose mean scores were significantly higher in the MRONJ group were the “functional limitation”, “physical pain”, and “social disability” (*p*-values: 0.004, <0.001, and 0.003) compared to those of the control group. 

Moreover, the MRONJ patients presented a poorer QoL. Indeed, the study group presented statistically significant higher scores in almost all the SF-36 items compared to the controls, namely in the “physical functioning”, “physical role”, “body pain”, “general health”, and “vitality” items (*p*-values: 0.001, 0.001, 0.013, 0.001, and 0.020, respectively). On the contrary, despite the lower mean scores in the remaining SF-36 items in MRONJ patients, specifically the “social functioning”, “emotional role”, and “mental health”, no statistically significant difference was detected (*p*-values: 0.082, 0.066, and 0.658, respectively). Additionally, although there was the absence of a significant difference in the latter items of the SF-36, which are mostly related to the mental component, the mean sub-scores of the HADS, both the anxiety and depression scores, were significantly higher in the MRONJ patients compared to the controls (*p*-values: 0.002 and 0.009, respectively). Indeed, symptoms of depression and anxiety (HADS-D and HADS-A sub-scores ≥ 8) were reported in 16 (64%) and in 4 (16%) MRONJ patients, while only 4 (16%) controls reported depression and none reported anxiety (data not displayed in the tables). With regard to the oral discomfort/pain, as expected, the mean score of the NRS was significantly greater for the study group compared to the control group (*p*-value: <0.001).

The Correlation analysis between the OHIP-14 total and sub-scores and the SF-36 items, HADS and NRS, is shown in Table 4 and Table 5. Interestingly, the total mean score of the OHIP-14 and the majority of the OHIP-14 items did not correlate with the SF-36 items, except for the “functional limitation” OHIP-14 item, which was negatively correlated to the “physical functioning” SF-36 item (*p*-value: 0.005), and the “physical pain” OHIP-14 item which was negatively correlated to the “general health”, “vitality”, and “mental health” SF-36 items (*p*-values: <0.001, 0.029, and 0.013, respectively) and positively correlated to the NRS mean score (*p*-value: <0.001).

When evaluating the relationship between HAD-A and HAD-D scores with the SF-36 items (Table 6), a negative and statistically significant correlation was found between depression score (HAD-D) and the “general health”, “vitality”, “social functioning”, and “mental health” SF-36 items (*p*-values: 0.046, 0.018, 0.004, and 0.003, respectively). This finding suggests that the scores of the majority of the SF-36 items related to the psychological aspect coherently varied with the depression score of the HADS and vice versa. Instead, HAD-A scores were only negatively correlated to the “bodily pain” and to the “mental health” SF-36 items (*p*-value: 0.006 and 0.031), meaning that patients with higher levels of pain and with psychological impairment (corresponding to lower “bodily pain” and “mental health” scores) also presented higher levels of anxiety. 

Comparison analysis among MRONJ patients divided according to their primary disease (osteoporosis vs. malignancy) revealed no statistically significant differences in any of the questionnaire scores, with the sole exception of the “physical pain” OHIP-14 item whose mean score was significantly higher among the osteoporotic patients (*p*-value: 0.009). Similarly, there were no differences even when comparing patients according to the MRONJ stage (stage I vs. stage II/III), except for the “physical disability” OHIP-14 item, and NRS means scores, which were statistically higher in patients affected by stage II/III MRONJ (*p*-values: 0.033, 0.012) (Table 7). Further, the scores and sub-scores of all the questionnaires were evaluated by separately comparing MRONJ stage I and MRONJ stage II/III with the controls (data not displayed in the tables). Interestingly, patients affected by MRONJ stage I (8, 32%) presented statistically significant higher scores only for the HADS-D and for the OHIP-14 physical pain sub-item (*p*-values: 0.049 and 0.008, respectively). On the contrary, OHRQoL, QoL, and psychological status were severely impaired in MRONJ stage II/III in comparison to the control group. Specifically, the study group presented statistically significant higher scores in almost all the sub-items of the OHIP-14 (functional limitation, physical disability, psychological discomfort, physical pain, and psychological disability) and in the OHIP-14 total scores (*p*-values: <0.001, 0.014, 0.009, <0.001, 0.001, and 0.008, respectively), as well as in the HADS-A and HADS-D sub-scores (*p*-values: 0.004 and 0.021). Similarly, patients with MRONJ stage II/III presented statistically significant lower scores in the majority of the SF-36 sub-items (physical functioning, social functioning, general health, vitality, physical role, and bodily pain) compared to the controls (*p*-values: 0.001, 0.043, <0.001, 0.008, 0.004, and <0.001, respectively). Overall, these findings suggest that MRONJ negatively affects the well-being of the patients, especially in the more severe stages of MRONJ.

## 4. Discussion

MRONJ is a severe drug-related adverse event developing in patients who have been treated with bone-modifying agents or anti-angiogenic medications, such as oncological patients with bone metastasis or patients affected by severe osteoporosis [37]. MRONJ is generally characterized by the presence of bone exposure in the oral cavity, recurrent infections, painful symptoms, and oral discomfort caused by altered oral functions that can negatively impair the perceived OHRQoL of patients [1,11]. Besides the primary diseases (either malignant or osteometabolic disease), which represent per se a great burden for the patients [38,39], MRONJ can also further aggravate their QoL due to its chronic course, often complicated by the difficulty in the treatments, poor response, and frequent recurrences [7,8,9,10]. Further, there is no general consensus on the therapeutic protocols, although both conservative and surgical treatments, especially with the adjunct of leukocyte–platelet-rich fibrin, have been proven to be effective [1,12,13,14,18,40]. In recent years, the attention to the QoL of the patients and to their psychological well-being has increasingly become of utmost importance in medical and dental research [41,42]. In this regard, patient-reported outcomes can help clinicians to tailor the treatments on account of the patient’s health status perception. However, to date, only a few studies have assessed the QoL of the MRONJ patients either as baseline evaluations [43] or as therapeutic outcomes [10,11,19,20,44,45]. Furthermore, no studies have specifically assessed the psychological profile of MRONJ patients. In the present case–control pilot study, MRONJ patients presented a significantly poorer OHRQoL and general QoL together with higher levels of anxiety and depression compared to the controls. 

The oral health self-perception was significantly affected in MRONJ patients, as demonstrated by the high average of the OHIP-14 total score (21.1 ± 12.7). In particular, the sub-items “functional limitation”, “physical pain”, and “social disability” of the OHIP-14 were significantly altered, meaning that MRONJ impacted the OHRQoL due to the limited oral function, painful symptoms, and discomfort in eating, which in turn may have caused psychological distress. These findings were in line with those of other studies. For instance, Miskad et al., by measuring the OHIP-14 scores before and after the diagnosis of MRONJ in a cohort of 34 oncological patients, found that MRONJ increased the severity scores from a mean of 3.56 to a mean of 16.53, resulting, therefore, in a worsening of the OHRQoL [43]. In another study, 35 stage III MRONJ patients experienced a great improvement in their OHRQoL after the surgical resection, with mean scores of the OHIP-14 drastically decreasing from 13.40 ± 9.27 at the baseline to 2.80 ± 3.60 after six months (*p*-value: <0.001) [45]. Interestingly, in both these two studies, the mean OHIP-14 total score at the baseline was slightly lower than the mean reported in our sample of patients. 

Similarly, Winter A et al. measured the OHRQoL of 36 MRONJ stage I/II patients by using the OHIP-49, and reported that patients’ perception of their oral health significantly improved after surgical treatment (*p* < 0.012) [44]. Altogether, these findings suggest that, although the OHRQoL of MRONJ patients may be severely compromised, surgical interventions are effective in improving patients’ OHRQoL regardless of the rate of clinical healing [44,45]. Furthermore, in our sample, although patients with stage II/III presented higher OHIP-14 mean scores compared to patients with stage I disease, no significant differences were found either in the mean OHIP-14 total scores or in the seven domains’ scores, except in the “physical disability” domain, as patients with MRONJ stage II/III complained more about the difficulty in eating due to interrupted meals, poor diet (*p*-value: 0.033), or severe pain (NRS *p*-value: 0.012). In this regard, Sato et al. also found no significant differences when comparing OHIP-14 scores among 33 unhealed patients with different MRONJ stages (mean OHIP total score of 10.12 ± 7.33 in stage I, 13.75 ± 11.61 in stage II, and 18.20 ± 14.96 in stage III) except for a worse sense of taste in stage III MRONJ (*p*-value: 0.027) [11]. Additionally, in the present study, MRONJ patients presented an overall poor oral health status compared to the control subjects, as demonstrated by the higher mean score of the DMFT and prevalence of periodontal disease, which could have further impaired their OHRQoL. These findings are in line with those from other studies suggesting that poor oral health may represent a pivotal risk factor for the development of MRONJ in predisposed patients or even a worsening of osteonecrosis [46,47].

The present study demonstrated that MRONJ patients’ QoL, as measured by the SF-36, was also negatively affected compared to that of the control group. Specifically, patients complained of impairments in their daily-life activities and work, of pain limiting their movements, of a poor self-perception of their general health, and of fatigue and tiredness. In two studies conducted on 20 and 30 MRONJ patients, authors assessed the QoL by using the SF-12 (the shorter version of the SF-36) and have likewise shown a poor QoL in their samples [10,48]. Specifically, Capocci et al. reported a significant difference in the physical and mental health among patients with different MRONJ stages, with MRONJ stage III showing the lowest scores of the SF-12 [48]. On the contrary, in our study as well as that by Tenore et al., no difference was detected between patients with different MRONJ stages [10]. This finding may be potentially explained by the small sample and the unequal distribution of cases among stages of MRONJ (8 in stage I, 16 in stage II, and 1 in stage III MRONJ). While in the present study, it is not possible to quantify the burden of the primary disease and MRONJ in reducing the QoL of the patients, there is evidence that oncological patients without MRONJ have a generally better QoL, especially for their function performance in daily occupations [5]. Furthermore, there is also strong evidence that surgical management of MRONJ is able to improve not only the oral health self-perception of the patients, but also the general QoL [20,24]. 

All the MRONJ patients (25, 100%) presented at least one or more systemic comorbidities in addition to the underlying osteometabolic or oncological disease requiring AR treatment compared to the controls (20, 80.0%), with a moderate statistically significant difference detected (*p*-value: 0.050). To note, although there was no statistically significant difference in terms of the prevalence of systemic diseases, a high percentage of MRONJ patients (32%) suffered from cardiovascular diseases, potentially due to the anticancer therapies’ short- and long-term cardiotoxic effect, which may have further aggravated the QoL of the patients [49].

With regard to the psychological profile, in the present study, MRONJ patients presented higher levels of anxiety and depression compared to the controls. In particular, almost 64% of the MRONJ patients suffered from depressive symptoms (HADS-D > 8), while only a small percentage (16%) had anxiety. To the best of the authors’ knowledge, there are no previous studies that have analyzed these two components with specific questionnaires. In this study, the HADS was chosen to evaluate the psychological status of the patients as it is one of the most used self-administered questionnaires for the evaluation of anxiety and depression both in the general population and in diseased patients. Of note, while the majority of the SF-36 items related to the psychological component, in particular the “mental health” item, did not differ between the groups, the HADS-A and HADS-D scores were significantly higher in MRONJ patients. Indeed, despite the good correlation with the HADS-A and HADS-D scores, the “mental health” item failed to detect any difference between the groups. Furthermore, no correlation was found between the OHRQoL (as measured by the OHIP-14) and the level of anxiety or depression (HADS-A and HADS-D). Altogether, these findings suggest that general questionnaires on both the OHRQoL and QoL (in this case, the OHIP-14 and the SF-36) may not be useful in the assessment of the psychological profile of MRONJ patients, as they may not have sufficient psychometric properties. Therefore, specific questionnaires are needed for a proper evaluation of the psychological impairment in MRONJ patients. Ultimately, the correlation analyses highlighted a generally poor correlation between the scores of the different questionnaires, thus suggesting that each of them is able to explore only some factors, and hence more tools should be used for a complete and thorough assessment of both the physical and mental status of the patients.

The findings of the present study might be considered in light of some limitations. First, the sample size of the patients included was small due to the pilot design of the study, and the patients included were both osteometabolic and oncological. Second, the control group comprised patients attending a dental clinic, potentially influencing the OHRQoL. Third, it is not possible to establish to what extent the poorer quality of life and the psychological impairment of MRONJ patients were dependent on the osteonecrosis of the jaws, on the underlying disease requiring AR therapy, or both. In this regard, future case–control studies should be conducted by comparing patients with the same baseline disease, with and without MRONJ. Furthermore, the subgroups contained different numbers of patients, so differences between groups may have potentially been missed. Finally, due to the absence of a validated Italian version of the MRONJ-QOL questionnaire [6], non-specific disease questionnaires were used to explore the oral health-related quality of life, the general health-related quality of life, and the psychological profile.

## 5. Conclusions

OHRQoL and general QoL were severely compromised in MRONJ patients in comparison with a group of controls matched according to age and sex. Additionally, psychological well-being was affected, especially because a high percentage of MRONJ patients (64%) suffered from depression. Specific questionnaires with psychometric properties should be used for the assessment of the mental component in MRONJ patients, as more generic instruments on the QoL can fail to detect the presence of mood impairment. Future research on a wide sample of patients is needed to test and validate a set of disease-based tools on QoL and psychological profiles to better guide treatment decision making.

## Figures and Tables

**Table 1:** Socio-demographic profile, body mass index, and risk factors in MRONJ patients and controls.

Demographic Variables	MRONJ	Controls	*p*-Value
	N° (%)	N° (%)	
**Gender** **Females** **Males**	21 (84.0) 4 (16.0)	21(84.0) 4 (16.0)	1.000
**Employment** Employed Not employed Retired	3 (12.0) 3 (12.0) 19 (76.0)	12 (48.0) 1 (4.0) 12 (48.0)	**0.046**
**Family situation** Married Not married Widowed	20 (80.0) 4 (16.0) 1 (4.0)	17 (68.0) 5 (20.0) 3 (12.0)	0.520
	**Mean ± SD**	**Mean ± SD**	***p*** **-value**
**Age (in years)**	72.3± 7.6	71.3± 6.9	0.367
**Education (in years)**	10.9 ± 5.2	8.5± 3.9	0.071
**Body mass index**	25.3 ± 4.3	26.7 ± 3.1	0.215
**Risk factors**	**N° (%)**	**N° (%)**	***p*** **-value**
**Smoking** Yes No	1 (4.0) 24 (96.0)	6 (24.0) 19 (76.0)	0.098
**Alcohol use** Yes No	1 (4.0) 24 (96.0)	5 (20.0) 20 (80.0)	0.189
**Systemic diseases**	25 (100)	20 (80.0)	**0.050**
Essential Hypertension	15 (60.0)	15 (60.0)	1.000
Hypercholesterolemia	2 (8.0)	6 (24.0)	0.247
Gastro-oesophageal reflux disease	1 (4.0)	1 (4.0)	1.000
Hypothyroidism	3 (12.0)	0 (0.0)	0.235
Diabetes	3 (12)	3(12)	1.000
Benign prostatic hypertrophy	0 (0.0)	2 (8.0)	0.490
Airway diseases	4 (16.0)	2 (8.0)	0.667
Previous myocardial infarction	0 (0.0)	1 (4.0)	1.000
Hyperthyroidism	2 (8.0)	0 (0.0)	0.490
Other cardiovascular diseases	8 (32.0)	2 (8.0)	0.074
Other systemic diseases	4 (16.0)	8 (32.0)	0.321

Legend: BMI = body mass index and MRONJ = medication-related osteonecrosis of the jaws. The difference was considered moderately significant 0.01 < *p*-value ≤ 0.05; strongly significant *p*-value ≤ 0.01.

**Table 2:** Clinical characteristics of MRONJ patients.

Clinical Variables	N (%)
**Total number of Patients**	25 (100)
**Oncologic patients**	17 (68.0)
*Solid tumor*	15 (60.0)
Breast	12 (48.0)
Prostate	3 (12.0)
Bone metastasis	13 (52.0)
*Multiple myeloma*	2 (8.0)
**Osteometabolic patients**	8 (32.0)
**Antiresorptive therapy (AR)**	
*AR monotherapy*	23 (92.0)
Zoledronate	2 (8.0)
Alendronate	5 (20.0)
Ibandronate	2 (8.0)
Denosumab	14 (56.0)
-XGEVA	12 (48.0)
-PROLIA	2 (8.0)
*Combined AR therapy*	2 (8.0)
Risedronate + PROLIA	1 (4.0)
Zolendronate + XGEVA	1 (4.0)
**Duration of AR (months)**	**Mean ± SD** 46.7 ± 44.3
**MRONJ site**	N (%)
-Mandible	16 (64.0)
-Maxilla	7 (28.0)
-Both	2 (8.0)
**MRONJ stage (AAOMS)**	
Stage I	8 (32.0)
Stage II	16 (64.0)
Stage III	1 (4.0)

Legend: AAOMS = American Association of Oral and Maxillofacial Surgeons; AR= anti-resorptive therapy; and MRONJ = medication-related osteonecrosis of the jaws.

**Table 3:** Comparison analysis of the questionnaires’ scores between MRONJ patients and controls.

Clinical Parameters	MRONJ	Controls	
	**Mean** **± SD**	**Mean** **± SD**	***p*** **-Value**
**OHIP-14 total score**	21.1 ± 12.7	11.0 ± 10.1	**0.003**
-Functional limitation	2.5 ± 2.1	0.9 ± 1.5	**0.004**
-Physical pain	4.6 ± 2.2	1.8 ± 1.1	**<0.001**
-Psychological discomfort	3.1 ± 2.3	2.7 ± 2.6	0.565
-Physical disability	3.1 ± 2.6	1.8 ± 2.4	0.074
-Psychological disability	3.4 ± 2.1	2.4 ± 2.5	0.107
-Social disability	2.5 ± 2.1	0.9 ± 1.5	**0.003**
-Handicap	1.9 ± 2.1	1.2 ± 1.5	0.154
**SF-36 items**			
-Physical functioning	44.0 ± 28.8	68.2 ± 17.3	**0.001**
-Physical role	37.0 ± 43.9	71.0 ± 21.0	**0.001**
-Bodily pain	47.8 ± 22.3	62.7 ± 18.3	**0.013**
-General health	31.7 ± 13.1	66.1 ± 11.9	**0.001**
-Vitality	52.0 ± 17.9	63.6 ± 16.2	**0.020**
-Social functioning	59.3 ± 24.8	63.6 ± 16.3	0.082
-Emotional role	51.8 ± 44.2	71.6 ± 28.1	0.066
-Mental health	59.9 ± 15.6	62.5 ± 24.1	0.658
**HADS-A**	6.1 ± 3.3	3.6 ± 1.9	**0.002**
**HADS-D**	8.4 ± 4.5	5.5 ± 2.8	**0.009**
**NRS**	3.2 ± 2.7	0.5 ± 0.6	**<0.001**

Legend: OHIP-14 = Oral Health Impact Profile-14; HADS-A = hospital anxiety and depression scale (anxiety subscale); HADS-D = hospital anxiety and depression scale (depression subscale); NRS = Numeric Rating Scale; MRONJ = medication-related osteonecrosis of the jaws; and SF-36 = Short Form 36 Health Survey Questionnaire. The difference was considered moderately significant 0.01 < *p*-value ≤ 0.05; strongly significant *p*-value ≤ 0.01.

**Table 4:** Correlation analysis between the OHIP-14 total score and sub-items of SF-36 in MRONJ patients.

Clinical Parameters	Physical Functioning		Physical Role		Bodily Pain		General Health		Vitality		Social Functioning		Emotional Role		Mental Health	
	**Rho**	***p*-Value**	**Rho**	***p*-Value**	**Rho**	***p*-Value**	**Rho**	***p*-Value**	**Rho**	***p*-Value**	**Rho**	***p*-Value**	**Rho**	***p*-Value**	**Rho**	***p*-Value**
**OHIP-14 total score**	−0.193	0.074	0.007	0.924	0.044	0.643	−0.025	0.222	−0.008	0.955	−0.014	0.891	−0.066	0.205	0.029	0.762
-Functional limitation	−0.045	**0.005**	0.015	0.158	0.003	0.821	−0.026	0.075	0.022	0.324	−0.026	0.101	−0.003	0.676	−0.001	0.929
-Physical pain	−0.027	0.106	0.005	0.624	−0.009	0.540	−0.065	**<0.001**	−0.054	**0.029**	−0.022	0.197	−0.012	0.124	0.039	**0.013**
-Psychological discomfort	0.033	0.156	0.001	0.990	0.026	0.206	0.009	0.661	−0.328	0.353	0.194	0.419	−0.011	0.311	−0.008	0.702
-Physical disability	−0.033	0.156	0.001	0.990	0.026	0.206	0.009	0.661	−0.032	0.353	0.019	0.419	−0.011	0.311	−0.008	0.702
-Psychological disability	−0.026	0.270	0.008	0.603	0.009	0.660	0.023	0.305	0.003	0.927	0.001	0.951	−0.002	0.801	−0.001	0.955
-Social disability	−0.012	0.496	0.007	0.535	0.019	0.220	0.027	0.112	0.178	0.491	−0.006	0.702	−0.014	0.101	−0.006	0.698
-Handicap	−0.014	0.365	0.006	0.569	0.005	0.705	0.005	0.720	−0.037	0.022	0.010	0.492	−0.013	0.705	−0.005	0.706

Legend: OHIP-14 = Oral Health Impact Profile-14; MRONJ = medication-related osteonecrosis of the jaws; and SF-36 = Short Form 36 Health Survey Questionnaire. The difference was considered moderately significant 0.01 < *p*-value ≤ 0.05; strongly significant *p*-value ≤ 0.01.

**Table 5:** Correlation analysis between the OHIP-14 total score and sub-scores with HAD-A, HAD-D, and NRS in MRONJ Patients.

Clinical Parameters	HAD-A		HAD-D		NRS	
	Rho	*p*-Value	Rho	*p*-Value	Rho	*p*-Value
**OHIP-14 total score**	0.634	0.477	0.119	0.851	1.329	0.130
-Functional limitation	0.250	0.067	−0.053	0.581	0.180	0.173
-Physical pain	0.115	0.480	−0.159	0.180	0.565	**<0.001**
-Psychological discomfort	0.010	0.939	0.056	0.668	0.050	0.779
-Physical disability	0.022	0.905	0.076	0.566	0.217	0.234
-Psychological disability	−0.001	0.994	0.060	0.639	0.134	0.443
-Social disability	0.060	0.675	0.070	0.496	0.100	0.476
-Handicap	0.171	0.186	0.068	0.463	0.080	0.523

Legend: OHIP-14 = Oral Health Impact Profile-14; HADS-A = hospital anxiety and depression scale (anxiety subscale); HADS-D = hospital anxiety and depression scale (depression subscale); NRS = Numeric Rating Scale; and MRONJ = medication-related osteonecrosis of the jaws. The difference was considered moderately significant 0.01 < *p*-value ≤ 0.05; strongly significant *p*-value ≤ 0.0.

**Table 6:** Correlation analysis between the SF-36 item scores and HAD-A, HAD-D, and NRS in MRONJ Patients.

Clinical Parameters	HAD-A		HAD-D		NRS	
**SF-36**	**Rho**	***p*** **-Value**	**Rho**	***p*** **-Value**	**Rho**	***p*** **-Value**
Physical functioning	−0.038	0.870	−0.143	0.408	−0.443	**0.038**
Physical role	−0.278	0.335	0.109	0.780	−0.103	0.799
Bodily pain	−0.310	**0.031**	0.027	0.780	−0.381	**0.013**
General health	−0.145	0.105	−0.132	**0.046**	0.052	0.565
Vitality	−0.183	0.076	−0.198	**0.018**	−0.044	0.672
Social functioning	−0.232	0.215	−0.303	**0.004**	0.019	0.920
Emotional role	−0.643	0.099	0.172	0.532	−0.001	0.997
Mental health	−0.216	**0.006**	−0.173	**0.003**	−0.001	0.988

Legend: HADS-A = hospital anxiety and depression scale (anxiety subscale); HADS-D = hospital anxiety and depression scale (depression subscale); NRS = Numeric Rating Scale; MRONJ = medication-related osteonecrosis of the jaws; and SF-36 = Short Form 36 Health Survey Questionnaire. The difference was considered moderately significant 0.01 < *p*-value ≤ 0.05; strongly significant *p*-value ≤ 0.01.

**Table 7:** Comparison analysis of the questionnaires’ scores between MRONJ patients according to their primary disease and MRONJ stage.

Clinical Parameters	Primary Disease			Stage AAOMS		
	Osteoporosis 8 (47%)	Malignancy 17 (68%)		Stage I 8 (32%)	Stage II/III 17 (68%)	
	Mean ± SD	Mean ± SD	*p*-Value	Mean ± SD	Mean ± SD	*p*-Value
**OHIP-14 total score**	26.3 ± 9.8	18.7 ± 13.4	0.124	16.5 ± 15.3	23.4 ± 11.1	0.281
-Functional limitation	3.6 ± 2.3	2.0 ± 1.8	0.115	2.1 ± 2.9	2.7 ± 1.6	0.613
-Physical pain	6.0 ± 1.4	3.8 ± 2.2	**0.009**	4.3.5 ± 3.1	4.7 ± 1.7	0.704
-Psychological discomfort	4.0 ± 2.2	2.7 ± 2.3	0.184	2.3 ± 2.2	3.4 ± 2.3	0.220
-Physical disability	3.1 ± 2.4	3.1 ± 2.7	0.951	1.5 ± 2.3	3.8 ± 2.4	**0.033**
-Psychological disability	4.5 ± 2.1	2.9 ± 2.0	0.106	2.6 ± 3.0	3.8 ± 1.5	0.318
-Social disability	3.0 ± 2.8	2.31.6	0.528	2.5 ± 2.8	2.5 ± 1.6	0.979
-Handicap	2.1 ± 2.6	1.8 ± 1.9	0.820	1.3 ± 1.5	2.3 ± 2.3	0.191
**SF-36 items**						
-Physical functioning	36.8 ± 31.1	47.4 ± 28.1	0.432	49.4 ± 37.6	41.4 ± 24.6	0.599
-Physical role	43.8 ± 47.7	33.8 ± 43.3	0.625	47.8 ± 41.1	32.5 ± 45.7	0.438
- Bodily pain	40.7 ± 27.3	51.1 ± 19.5	0.354	63.4 ± 27.2	40.5 ± 15.6	0.053
-General health	33.8 ± 12.1	30.7 ± 13.8	0.568	29.3 ± 14.9	32.8 ± 12.5	0.582
-Vitality	58.12 ± 28.14	49.1 ± 10.3	0.406	56.2 ± 20.5	50.0 ± 16.8	0.467
-Social functioning	54.1 ± 33.3	61.6 ± 20.5	0.591	65.5 ± 34.4	56.4 ± 19.3	0.503
-Emotional role	45.7 ± 46.9	54.7 ± 44	0.655	54.0 ± 43.4	50.8 ± 45.8	0.871
-Mental health	66.5 ± 24.7	56.8 ± 8.2	0.313	62.5 ± 16.2	58.7 ± 15.6	0.591
**HADS-A**	7.0 ± 4.7	5.6 ± 2.3	0.465	5.5 ± 3.5	6.3 ± 3.2	0.569
**HADS-D**	7.5 ± 5.2	8.8 ± 4.4	0.544	8.12 ± 4.6	8.5 ± 4.5	0.842
**NRS**	3.1 ± 2.8	3.2 ± 2.7	0.928	1.5 ± 1.7	4.0 ± 2.7	**0.012**

Legend: OHIP-14 = Oral Health Impact Profile-14; HADS-A = hospital anxiety and depression scale (anxiety subscale); HADS-D = hospital anxiety and depression scale (depression subscale); NRS = Numeric Rating Scale; MRONJ = medication-related osteonecrosis of the jaws; and SF-36 = Short Form 36 Health Survey Questionnaire. The difference was considered moderately significant 0.01 < *p*-value ≤ 0.05; strongly significant *p*-value ≤ 0.

## Data Availability

The data presented in this study are available on request from the corresponding author. The data are not publicly available due to privacy reasons.
